# The unforeseen future: Impacts of the COVID‐19 pandemic on home video‐EEG telemetry

**DOI:** 10.1111/epi.17473

**Published:** 2022-12-18

**Authors:** Franz Brunnhuber, Jeremy D. Slater, Sushma Goyal, Devyani Amin, Joel S. Winston

**Affiliations:** ^1^ Department of Clinical Neurophysiology King's College Hospital London UK; ^2^ Alliance Family of Companies Irving Texas USA; ^3^ Evelina London Children's Hospital, Guy's & St Thomas' NHS Foundation Trust London UK; ^4^ Department of Basic and Clinical Neurosciences, Institute of Psychiatry, Psychology and Neuroscience King's College London London UK

**Keywords:** ambulatory video‐EEG telemetry, home video‐EEG telemetry, remote diagnosis, video‐EEG telemetry

## Abstract

The coronavirus disease 2019 (COVID‐19) pandemic had widespread impact on health care systems globally—particularly services arranged around elective admission and attendance such as epilepsy monitoring units and home video‐EEG telemetry (HVET). Here, we review the ongoing impacts of the pandemic on HVET services among several different providers who used different initial models of HVET. We discuss the features of HVET that led to success in providing continued diagnostic services to patients with epilepsy and related disorders and through retrospective audit of our services demonstrate the high diagnostic yield of HVET. We reflect on this unforeseen future and its implications for other diagnostic techniques and approaches.

**Key points**

- The coronavirus disease 2019 (COVID‐19) pandemic provoked significant disruption for inpatient diagnostic testing for patients with suspected seizures.
- Home video‐EEG (electroencephalography) telemetry services were less disrupted than those that depended upon inpatient admission.
- Home video‐EEG telemetry showed good capture rates for interictal abnormalities and clinical events.
- This is in keeping with a wider trend toward remote diagnosis, management, and care of patients with epilepsy and other conditions.

## 1 INTRODUCTION

In 2019, we surveyed the state of home video‐EEG (electroencephalography) telemetry (HVET)^1^ and noted that although it was less expensive than inpatient video telemetry (VT) within multiple health care systems,^2^, ^3^ it was noninferior in terms of data quality and rate of event capture for adults.^4^, ^5^ We noted that HVET was highly scalable, based upon the centralization of expertise and that it obviates the need for some components of tertiary health care infrastructure. Due to this scalability and cost‐effectiveness we anticipated that the steady growth trajectory of HVET we were seeing within our own services would continue over the next few years.^1^ We did not anticipate a global pandemic and the complex effects that this would exert on health care systems.^6^, ^7^, ^8^

Others hypothesized early during the pandemic that HVET might fulfill a role, given the pressures befalling inpatient health care services.^9^ Here we briefly reiterate and update the distinct models of HVET^1^ and proceed to review the experiences of our three different centers. Each possessed the advantage of significant experience with HVET before the pandemic, and we would not expect that these experiences reflect those of all tertiary epilepsy‐monitoring units. We explore what worked well at each center and identify common themes and distinctions. We consider whether the changes we characterize represent a transient peri‐pandemic phenomenon or a lasting shift in this form of health care provision. Finally, having described this “unforeseen future” of HVET, we briefly review some new home diagnostic technologies relevant to epilepsy and address whether the changes that have prompted HVET uptake will drive the adoption of these related technologies.

## 2 MODELS OF HVET

We have previously reviewed the various approaches adopted to HVET.^1^ Here, we propose a subtly altered terminology to that we have suggested previously (Table 1). We maintain the term “ambulatory HVET” for the approach in which the study proceeds after connection with no further in‐person attention from hospital staff. We suggest “daily supervised HVET” for the model we referred to previously as “supervised HVET”; in this approach, a physiologist attends the patient's home daily during the study, performs data quality checks, and may transport the data acquired thus far back to the hospital. The modifier “daily” implies the typical frequency of supervision, as the data are normally unavailable for review between technician visits. A final approach is “cloud supervised HVET”; in this version, the data are live‐streamed via the patient's home internet connection or a mobile data connection to a secure cloud service and can be reviewed in real‐time. This allows online data‐quality checking and review and permits the study to be considered a monitoring study for reimbursement purposes in the United States (see Section 3.3, Stratus). Within the National Health Service (NHS; the UK health care system), any of the three models warrant the standard tariff for VT.

**TABLE 1:** Proposed nomenclature of HVET study types and comparison between these approaches

	Ambulatory HVET	Daily supervised HVET	Cloud supervised HVET
Summary	EEG connected and no further intervention until return of equipment	EEG connected; daily visits from physiologist to check electrodes/data quality and potentially download data	EEG connected and recording equipment streams data as it is acquired to cloud storage for remote access by care team
Electrode attachment	Home or hospital	Home or hospital	Home or hospital
Visits from physiologist	None after electrode attachment	Typically once daily	None after electrode attachment
Data stored	On device until return to hospital	On device until physiologist's visit; can then be physically transferred to hospital	In secure cloud storage facility
Data quality check	None after initial setup	Daily during visit	Can be monitored regularly
Data pruning/review	After equipment return	After daily data transfer or after equipment return	During study
Location of data reviewer	Typically hospital	Typically hospital	Hospital or remote location (subject to governance constraints)

Abbreviations: EEG, electroencephalography; HVET, home video‐EEG telemetry.

## 3 THREE HVET SERVICES DURING THE COVID‐19 PANDEMIC

### 3.1 King's College Hospital (KCH)

**FIGURE 1:**
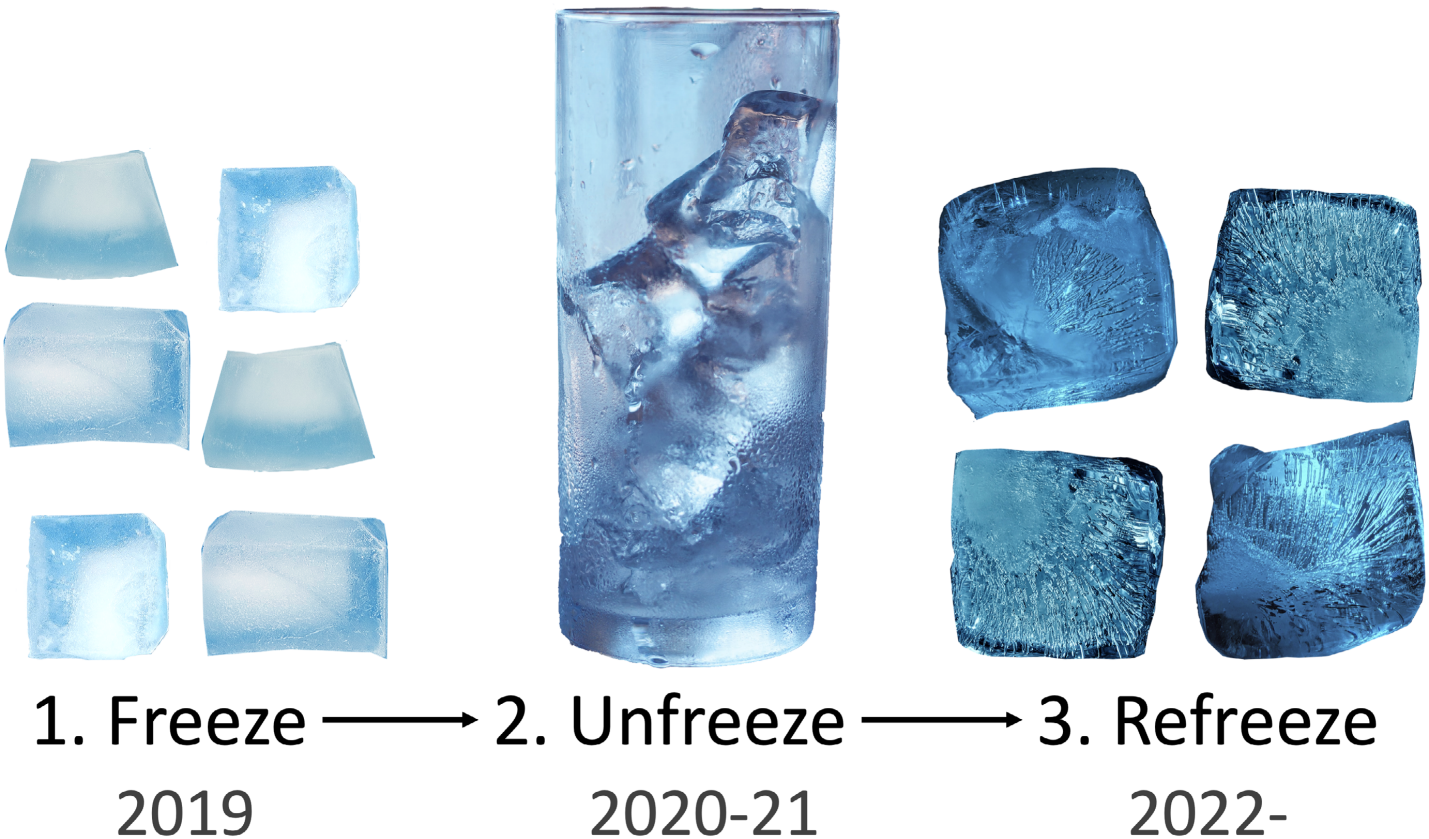
A model of organizational change (after Lynda Gratton; “Redesigning work,” 2022). Organizational change from pre‐existing structures (“freeze”) is precipitated by shocks to the system (in the analogy, “unfreezing”). The induced instability permits a process of change and the eventual formation of new structures with different characteristics (e.g. fewer larger ice cubes; here with the same total volume) through re‐freezing.

Inspired by Lynda Gratton's Redesigning Work^10^ (with a model based upon Kurt Lewin's model of organizational of change^11^) we consider the impact of the pandemic in terms of a Freeze‐Unfreeze‐Refreeze model. We apply these principles to a diagnostic service within the NHS at our South London teaching hospital. The pre‐pandemic situation showed clear structures, orientations, and resources as symbolized by the six frozen ice cubes (Figure 1). Due to the monumental changes triggered by the pandemic, structures begin to dissolve (“unfreeze”). This allowed new ways of thinking, practicing, and delivering health care to be explored. The substance (water) remains the same, though in a new aggregate (liquid). The “refreeze” stage shows the new forms, new orientations, and new entities that emerge, symbolized by the four bigger ice cubes. The adoption of this model is illustrative, rather than prescriptive, and it can be expected that with the pandemic ongoing, the “refreeze” perhaps remains incomplete and will certainly vary from center to center.

KCH was at the epicenter of the of the initial coronavirus disease (COVID) outbreak in the United Kingdom (UK), and the pandemic had widespread effects here, triggering redeployment of staff, and cancellation of some elective inpatient and outpatient services while resources were redeployed to care for sick inpatients. This had a huge impact on the KCH Department of Clinical Neurophysiology. Here, we consider the effects only upon the VT services.

#### 3.1.1 KCH inpatient VT

Our pre‐pandemic level of activity was 4.6 patients per week. With the first lockdown (announced in mid‐March 2020) the hospital beds normally reserved for inpatient VT were needed for the treatment of patients with coronavirus disease (COVID) and the service ceased entirely. By June 2020 we were encouraged to restart the service and were able to admit 2.5 patients per week until the end of the year. However, with a substantial further wave of infections from late December into March, the VT beds were again used for patients with COVID (January to March 2021). Limited re‐opening permitted admission of 2.8 patients/week from April to December 2021, but a further significant pandemic wave as restrictions were lifted led to VT bed closure again for 2 months. In March to April 2022 we were reopened at a rate of 2.1 patients per week, amounting to 46% of patient throughput compared to pre‐pandemic levels.

Elective inpatient admission for VT during the pandemic (when it was even feasible) proved more challenging for patients than was historically the case. They were asked to self‐isolate for periods between 3 and 14 days (depending upon local policy during different phases of the pandemic). Many patients expressed concern about the prospects of contracting COVID in the inpatient setting. Some patients had stays canceled due to suspected or proven severe acute respiratory syndrome coronavirus 2 (SARS‐CoV‐2) infection. A substantial number of admissions were canceled or postponed on relatively short notice, in some cases even after the patient had undergone self‐isolation due to the dynamic situation and pressure on hospital inpatient services.

#### 3.1.2 KCH HVET service

**FIGURE 2:**
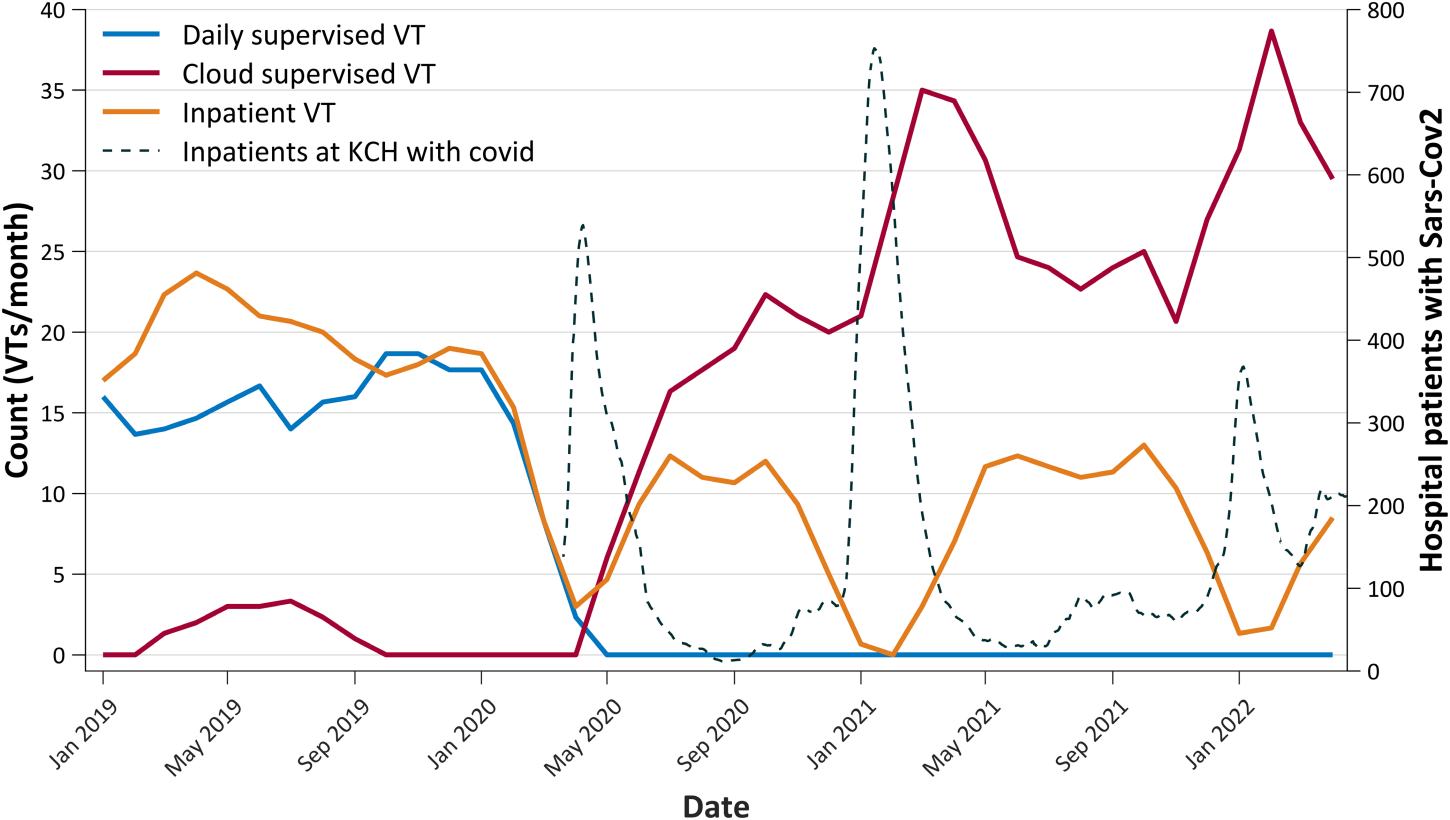
Video telemetry (VT) studies/month at King's College Hospital (KCH) by type (3‐monthly rolling averages) and COVID bed occupancy. May 2019 represents our trial of a cloud‐based HVET system. The peaks in Spring 2020, Winter 2021 and Winter 2022 reflect the waves of high infection rates and high inpatient bed occupancy at KCH (gray dashed line; right y‐axis); during those periods inpatient VT was almost completely precluded, whereas at the latter stages of the pandemic, cloud‐based HVET proved resilient and continued, even allowing an increase in activity (seen after the large waves of infection and covid‐related hospital admissions in the winters of 2021 and 2022). Covid bed occupancy data is NHS Trust‐level data from https://coronavirus.data.gov.uk and are plotted daily with a rolling average of 1 week.

In the year before the pandemic we recorded HVET studies for 4.3 patients per week. This predominantly used our daily supervised HVET model,^1^ although from December 2019 we had started to evaluate and had sought local regulatory approval for a cloud‐supervised HVET system. COVID regulations called for a significant reduction in direct contact between staff and patients, and daily supervised HVET was deemed to rely on excessive staff/patient contact due to the requirement for home visits. This service was, therefore, replaced by the cloud supervised HVET approach. The cloud‐based service became the main VT service model from June 2020. In the first 7 months we were able to see 7.8 patients per week (>80% activity increase over pre‐pandemic levels). During the last period in which inpatient VT was precluded (January to March 2022) we were able to more than double our weekly activity (235% increase) for several weeks (Figure 2).

The flexibility and scalability of the cloud‐based HVET service has allowed us to establish another two services since January 2021: remote VT cloud‐supervised recordings for inpatient studies in surrounding district hospitals and 24‐h HVET recordings for patients who had failed sleep studies in the hospital (i.e., HVET with the primary intention of capturing sleep EEG rather than paroxysmal events).

In 2019, our service saw no significant difference in demand (referrals) for HVET compared to inpatient VT. By 2021, demand for inpatient tests dropped by 33%, whereas demand for HVET increased by 78%. This trend continued in 2022, leading to an 84% demand increase for HVET, whereas demand for inpatient studies dropped by 48% compared to pre‐pandemic levels.

Naturally, observing these substantial shifts in our practice raises the question of whether the nature of HVET studies undertaken at KCH has changed over this period. We have audited this, using searches of electronic medical records. Over the period from January 2019 to July 2022 a total of 835 HVET studies were undertaken. Demographic data (patient gender/age) were extracted as well as study duration. The clinical question asked was categorized into one of seven categories (1. Event capture, 2. Syndromic classification, 3. Seizure quantification, 4. Sleep disorder, 5. Pre‐intervention [e.g., pre‐surgical assessment, consideration of cannabidiol], 6. Background assessment, 7. Electrical status epilepticus during slow‐wave sleep). Report conclusions were used to categorize the presence of nonspecific abnormalities, epileptiform abnormalities, habitual event capture (and their nature; e.g., epileptic, nonepileptic, sleep‐related, uncertain, or “other”), and nonhabitual event capture (and their nature; e.g., epileptic, subclinical seizure, or “other”). Finally, whether the question asked was answered by the study was coded. Not every study undertaken could be automatically matched to a full‐text report due to errors in the way that reports were uploaded onto the electronic medical record, and not every study was accompanied by a clearly documented question. In total, a specific question could be categorized in 674 cases (80.7%) and full‐report text was available in 756 (90.5%). Pandemic “onset” was defined as March 23, 2020 (the announcement of the first UK lockdown and the date from which KCH clinical services were radically reorganized). A total of 208 studies analyzed were “pre‐pandemic” and 628 were “peri‐pandemic.”

Some shifts in demographics (gender and age) for patients undergoing HVET at KCH occurred between the two phases. The gender balance did not change significantly (61.1% female pre‐pandemic to 57.9% female peri‐pandemic) but patients’ age at the time of the stage significantly decreased (from median 33.2 to 27.2 years; *p* < .01). There was a small decrease in median study duration peri‐pandemic (from 44.5 h to 44.1 h), very likely due to the new overnight HVET service. The overwhelming majority of HVET requests were for “event capture” (85% overall). Peri‐pandemic there was a decrease in the use of the generic “event capture” indication (decreased by 8.9%) and small increases in other categories (the main change being an increase in “background capture” [1.2 → 4.8%, again likely a function of the new overnight HVET service]).

In terms of outcomes from HVET, a high proportion of questions were answered overall (61.3%). This increased slightly from pre‐ (55.4%) to peri‐pandemic (63.5%); an effect of borderline significance ($\chi_{\mathrm{df}=1}^{2}$  = 4.0; *p* = .046). A likely contribution stems from the increased proportion of requests for background (as opposed to event) capture in the form of requests for overnight HVET. There was an increase in the proportion of reports capturing interictal abnormalities, both nonspecific (49.2%–59.2%) and epileptiform (36.9%–48.1%). There was no substantial change in the overall rate of habitual event capture between the pre‐pandemic period (57.4%) and peri‐pandemic (55.1%). No individual event subtype showed significant change, although there was a numerical increase in the proportion of studies capturing epileptic seizures (15.4% increasing to 19.6%) and a decrease in the proportion of studies capturing events categorized as nonepileptic (28.2% decreasing to 24.4%).

#### 3.1.3 Lessons learned

Our daily supervised HVET service model did not prove resilient in the context of the pandemic and its necessary regulations. A cloud‐supervised HVET service showed both flexibility and scalability to accommodate new services and patient needs. We noticed a significant increase for our VT services in total, but this masks a change in which demand for inpatient recordings dropped and demand for HVET increased. Not infrequently were patients reluctant to commit to a hospital admission, as this required a significant period of isolation before admission. Furthermore, the switch to cloud‐supervised HVET allowed an increase in staff (both medical and physiologists) working away from the hospital site; this allowed better social distancing for those in the workplace and safer working conditions. Informally, patients reported that they liked the cloud‐supervised approach and that data could be checked remotely if they had concerns; a mobile number was provided for queries arising at any time. We do not yet know whether the described trends will continue, but it appears that the pandemic was a clear catalyst for telemedicine, at least transiently. A subset of patients who required more prolonged assessment for event capture (longer than 4–5 days) or antiseizure medication reduction continued to wait for inpatient VT, with effects on service delivery that are still ongoing.

### 3.2 Stratus

In the years leading up to the pandemic, major changes occurred in the American medical establishment impacting ambulatory EEG monitoring. The complete revision of the Current Procedural Terminology (CPT) codes covering extended video‐EEG recording effectively acknowledged the existence of ambulatory EEG as a diagnostic tool. Prior to this revision payor denials for reimbursement categorizing the diagnostic procedure as “experimental” were not uncommon. There were significant reimbursement difficulties, with some payors taking more than a year to add the new code set. The diagnostic outcomes of a 10 000‐patient cohort were published in 2019 alongside an accompanying editorial that directly stated: “There is currently underutilization of ambulatory techniques in evaluating patients with episodic neurological, in addition to non‐neurological conditions.”^5^, ^12^

These changes, along with continuing advances in technology and the more general overall push for health care in the United States to move to an outpatient setting, resulted in a progressive increase in the number of ambulatory video‐EEG studies performed. Limits to that increase came from hospital systems concerned about reduced inpatient volumes, and a roughly 30% decrease by payors in the reimbursement for the technical portions of the service.

On March 11, 2020, the World Health Organization declared coronavirus disease 2019 (COVID‐19) a pandemic. By March 18, the Centers for Medicare & Medicaid Services (CMS) announced that all elective surgeries and nonessential medical, surgical, and dental procedures be delayed. Over the next several months, elective admissions were suspended at hospital systems across the United States, including those for epilepsy monitoring units (EMUs). Consequently, inpatient epilepsy diagnostic evaluations and surgical interventions for epilepsy came to a near total halt. With mounting numbers of patients facing indefinite delays in diagnosis, physicians and hospital systems turned to ambulatory video‐EEG monitoring as the only alternative.

**FIGURE 3:**
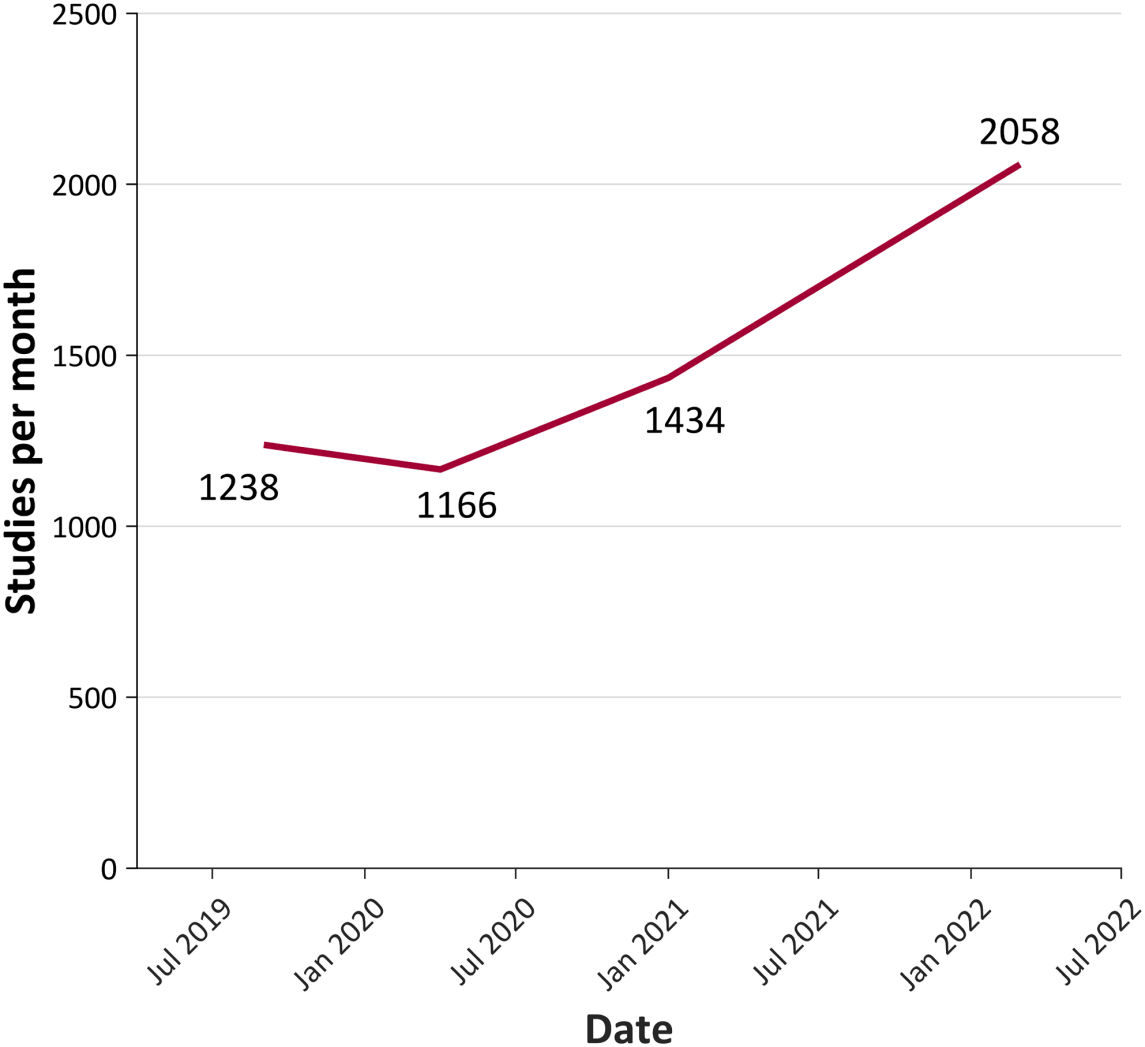
Increase in demand for Stratus Neuro home video‐EEG (electroencephalography) telemetry (HVET) studies in the United States over the period of the pandemic.

Many of these new clients specified that “this is temporary—we will only be using ambulatory services until we can reopen our EMU.” Despite this warning, with the waning of pandemic numbers and the restoration of EMU services, the number of orders for ambulatory studies only continued to increase (Figure 3). The likely explanation is multi‐factorial. Patients generally prefer diagnostic testing in their home as opposed to entering a hospital.^13^, ^14^, ^15^ The bias against hospitalization was likely accentuated given the large number of COVID‐19‐positive patients in the hospitals during the peak of the pandemic and the fear of exposure. The general increase in telehealth visits with pandemic restrictions and the relaxation of payor restrictions on reimbursement for such visits also likely contributed to increased acceptance of in‐home video‐EEG studies. Finally, physician exposure (in many cases for the first time) to the improved technology that allows state of the art recordings broke down longstanding biases regarding the technical quality.^16^ Consequently, it appears the “genie cannot be returned to the bottle” and HVET will be an increasingly important diagnostic test now and into the future.

### 3.3 Evelina London Children's Hospital

Evelina London Children's Hospital is a teaching hospital situated adjacent to St Thomas' Hospital, which looked after the majority of adults with COVID‐19 in the London Borough of Lambeth. At the peak of the pandemic, up to 450 patients with COVID‐19 were admitted. The Evelina pediatric intensive care unit (PICU) and its nurses were re‐deployed to help with the overflow of some these adult patients. Children on the PICU were moved to a temporary facility with reduced nursing and medical capacity. The Evelina became one the first centers to report children with pediatric inflammatory multisystem syndrome temporally related to SARS‐CoV‐2 (PIMS‐T),^17^ as it was a regional tertiary referral center.

#### 3.3.1 Evelina London Children's Hospital—VT service

Given the re‐deployment of nurses and junior doctors, the inpatient VT facility within the neurology ward was also closed during the two peak periods of the pandemic. Inpatient VT is considered an elective procedure, and in most centers, particularly in tertiary centers, all elective work was suspended to allow for reallocation of resources to the increasing numbers of patients being admitted with COVID‐19. Our approach at the Evelina London Children's Hospital was different from other centers. The pediatric neurophysiology service is based in a dedicated children's hospital, and the hospital's medical leadership decided to continue to offer essential services for children while ensuring safety of the staff and preparing ourselves to help the adult center if necessary.

In March 2020, we determined that EEG and VT for some of the referred children was not an “elective” but an “essential” test, as delays in diagnosis, misdiagnoses, and delays in appropriate management have a high likelihood of impacting long‐term outcome in children.^18^ Between March and June 2020, we triaged referrals into three categories using a scale formulated by a pediatric neurology colleague:

1. Time‐critical treatment, if delayed has a high likelihood of impacting long‐term outcome. Diagnostic tests that are likely to alter immediate management to either prevent further morbidity or to improve long‐term outcome.
2. Treatment ideally given at a specified time, but some delay is reasonable, for example, evidence base is less secure for improved outcome, or evidence is lacking that delay is harmful or the child is clinically stable. Includes diagnostic tests that may alter management in the longer term but are unlikely to result in a time‐critical change in management that will significantly affect outcome.
3. Non‐urgent/no time pressure. May provide diagnostic or prognostic information but this is not expected to alter treatment approach.

Seventy‐nine families on the waiting list for VT and triaged as above were contacted by phone; 77% said they were willing to come for the test and travel to our hospital (located in central London) before traveling home for HVET. Seventeen families (all category 2–3) asked for the test to be postponed, citing COVID‐19 as the reason. Parental feedback was taken after the test; 89% of responders said they felt safe and would return if needed for another test during the pandemic. Ninety‐four percent of parents found the ambulatory HVET kit easy to use, with none or only minor issues encountered. Hospital protocol for personal protective equipment, risk assessment for staff, and infection‐control procedures were undertaken. Only one carer was allowed to attend and parking was available. Based on guidance from the British Society for Clinical Neurophysiology,^19^ hyperventilation was not performed.

Pre‐pandemic the Pediatric EEG service at Evelina London performed ~1000 EEG studies per year on average, of which 30% were urgent in‐patient EEG studies, consisting mainly of portable bedside video‐EEG. Over three hundred VT studies were undertaken in 2019. The inpatient VT service started in 2009, with dedicated beds on the neurology ward. After the introduction of our HVET service in 2015 (based on ambulatory HVET), we witnessed a seismic shift in the proportion of HVET vs inpatient VT; by 2019, 71% of telemetry studies were performed as HVET. HVET demand had increased consistently over the years from referrers and parents of children undergoing the test who preferred this over an in‐patient admission. Although the indication for the majority of the HVETs was for diagnostic purposes (1‐ to 3‐night recordings), over time we also built experience in offering HVET to pre‐surgical patients in whom the seizure burden was high, as this did not require a longer hospital stay with drug reduction. Our non‐collodion method^20^, ^21^ requires the child to visit the hospital only once for connection, during which a baseline recording including activation procedures and clinical history is obtained. After the test is completed (24–72 h), the parents remove and discard the disposable electrodes and return the equipment via our dedicated service courier.

**FIGURE 4:**
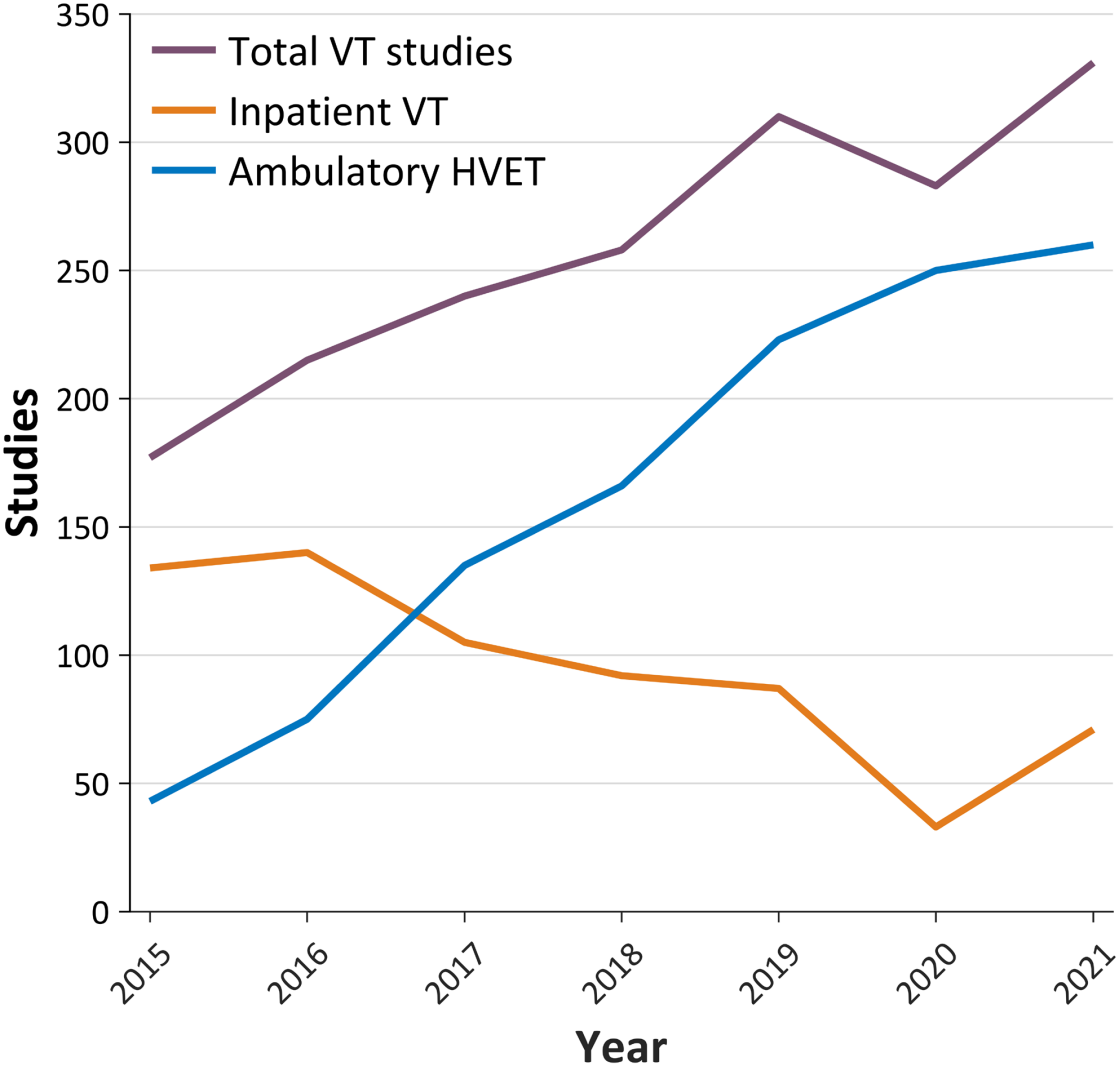
Growth in home video‐EEG (electroencephalography) telemetry (HVET) studies at the Evelina London Children's Hospital. Number of studies each year of each type are plotted, along with the total number of video telemetry (VT) studies (yellow line). Note the dip in inpatient studies during 2020, which was largely offset by increasing the number of HVET studies undertaken.

Overall, there was only a 10% reduction in our year‐on‐year activity in 2020; the VT service was maintained largely by increasing the number of HVETs performed during the pandemic (Figure 4). The main loss was our inability to offer inpatient VT for those children who required drug reduction. The ability to maintain the service during the pandemic has led to reduced waiting times, which have been consistently below the national average. The validation of this strategy came from the International League Against Epilepsy and the International Federation of Clinical Neurophysiology in their consensus statement in 2021, who reiterated that long‐term video‐EEG monitoring is an essential service and have called for continuing function of EMU during future emergency situations, such as the COVID‐19 pandemic.^22^

Our experience confirms that this can also be achieved by integration of HVET with existing inpatient VT units. In addition, we were able to continue the pre‐surgical assessment of a select group of children by using HVET, which helped to prevent a delay in their epilepsy surgery pathway.

We have retrospectively audited the Evelina HVET service during a period similar to that of the audit of KCH studies described earlier (January 2019 to December 2021). Again, studies undertaken before March 23, 2020, were considered pre‐pandemic and those after that date categorized as peri‐pandemic. Study identification used a search of the EEG database to identify HVET studies; full‐text reports were then exported and data extracted semi‐automatically, with manual classification of test indication and outcomes (both with the same categories as KCH). A total of 723 reports were identified from the relevant period, representing 99% of the relevant studies undertaken.

Patient demographics (age/gender) were unchanged over the period audited. Study duration was extracted from the EEG database directly and was available for 84.1% of studies. The overall median study duration was 33.9 h and there was a clear increase from pre‐pandemic (median 31.1 h) to peri‐pandemic (35.4 h). This reflects a policy change over time that is independent of the pandemic, whereby studies are increasing triaged for two day rather than one day recordings. Study indications were identified in 98.8% of reports. Most studies were indicated for “event capture” but this decreased slightly as a proportion of referrals from pre‐pandemic (75.1%) to peri‐pandemic (69.7%). An increase of similar magnitude was seen in referrals for pre‐intervention assessment (e.g., pre‐surgical VT or before starting cannabidiol), which increased from 4.8% of referrals to 10.0%. Other referral categories were essentially unchanged.

Studies were judged to have answered the referral question in a very high proportion of cases, which did not change from pre‐ to peri‐pandemic (74.4% and 74.6%, respectively). The proportion of reports mentioning nonspecific interictal abnormalities increased from 49.2% to 57.7%, whereas there was no change in the proportion of studies finding epileptiform abnormalities (61.6% vs 61.8%). Habitual event capture was also similar between the pre‐pandemic and peri‐pandemic periods (68.8% vs 70.5%), and there was no event subtype for which the proportion captured varied significantly. A small nonsignificant increase in the proportion of studies capturing epileptic seizures (31.0% pre‐pandemic vs 34.7% peri‐pandemic) was noted.

#### 3.3.2 Evelina Children's Hospital—Urgent video review

There have been other unexpected consequences of our working practice during the pandemic. To triage urgent EEG referrals we asked for all urgent referrals to be consultant‐to‐consultant with the aim of increasing the pre‐test probability and test yield.^23^ In addition, we requested that home video (largely smartphone‐derived) clips be sent to help triage referrals, especially when events such as spasms were referred. During the height of the first wave of the pandemic, the hospital funded an NHS‐approved video review app (vCreate; https://www.vcreate.tv/) through which families could securely upload videos. Over the next year we started receiving nonurgent video review referrals from pediatric neurologists. The demand for this has increased, and in 2022 we formalized this with a new video‐review service. Referred videos are reviewed in a virtual multi‐disciplinary team meeting (MDT) meeting for consensus opinion. A formal report is then generated by the clinical neurophysiologist for clinical governance and documentation of the discussion. On many occasions this has averted unnecessary additional tests such as VT for children with clinically obvious nonepileptic events or has helped prioritize testing where pre‐test probability was high, for example, in infantile spasms. We are in the process of auditing this new service.

#### 3.3.3 Evelina Children's Hospital—Remote working patterns

All our EEG and VT meetings including epilepsy surgery MDT transitioned to virtual formats in spring 2020. This resulted in an increase in attendance with an inclusion of a wider audience including trainees and outreach clinicians, who had previously been unable to commute to attend the in‐person meeting. Virtual (or at least hybrid) MDTs are likely to continue beyond the pandemic.

To allow for flexible working patterns for staff we had by chance purchased extra remote review licenses in February 2020, just prior to the pandemic. This allowed us to review and report the HVET studies remotely in the pandemic, particularly for those staff who were shielding or isolating at home but were well enough to work. Over the last year we have doubled the number of remote review licenses allowing each member of staff to work from home once a week.

## 4 DISCUSSION

The experiences of our three centers over the pandemic show some significant similarities, despite the different populations served and provider/reimbursement models. There was a striking resilience to HVET, sometimes after modifications from pre‐existing practices. In contrast, inpatient VT was not robust; initially this was a function of loss of beds and closure of elective services but later in the pandemic a greater mix of factors contributed including patient preference for avoidance of hospital facilities.

The main modification made at KCH that helped to improve the robustness of HVET was the move to a cloud‐based approach (Table 1). This reduced the requirement for face‐to‐face contact and was much more readily compatible with the flexible working patterns that medical and technical staff adopted during the waves of high infection. In the United States, the cloud‐based approach was a pre‐requisite for reimbursement as a monitoring (as opposed to ambulatory) procedure. At KCH, this switch to cloud‐based EEG led to further service developments including the improvement of provision of inpatient EEG at referring hospitals. The cloud‐based approach for remote hospital studies allowed provisional reports to be communicated live. It further enabled clinical decisions on how best to proceed with studies after initiating a recording (e.g., trials of drug therapy, diagnosis of status epilepticus, and decisions to extend studies into prolonged recordings).

The comprehensive audits of clinical reports from KCH and Evelina Children's Hospital described earlier confirm the high utility of HVT for habitual event detection (with 55%–70% of studies capturing habitual events), interictal anomaly detection (40%–60%), and successfully answering the clinical question asked (61%–75%). Although some small changes were noted between the pre‐pandemic and peri‐pandemic time periods audited, these differences were generally of small magnitude and often reflected obvious changes in clinical practice (e.g., the launch of a new overnight HVT service at KCH, or the shift to performing some pre‐surgical assessment remotely in both centers). Nonetheless, it is impossible to infer from these data that the pandemic did not change the patient mix referred for studies, and the patterns of change described varied between the two centers, suggesting a complex mix of factors contributed.

As described earlier, we see that one perspective on the pandemic is that it formed a facilitator of organizational change. The “melting” of existing structures allowed new practices to form, and some new structure is now evident. Within our services, despite the removal of almost all pandemic‐related restrictions for several months, there is no significant reversal of the increased utilization of HVET. It appears that its predicted benefits during the pandemic^9^ have been proven true, and the upshot is that its place in the array of epilepsy‐related diagnostic testing is secured and likely expanded.

The expansion of HVET services we have described implies one component of the future of HVET, whereby it replaces some other types of tests that are traditionally undertaken on an inpatient basis. It is interesting to speculate on what else the future of HVET might hold. A further expansion might include the use of the cloud‐based HVET model to facilitate the provision of urgent remote inpatient services for patients at remote hospitals (the “remote VT” model mentioned in Section 3.1.2). Another potential expansion stems from the use of HVET as an adjunctive study—for patients who have undergone inpatient VT (perhaps including antiseizure medication reduction) in whom habitual events have not been captured, the patient can be discharged with an HVET study established to potentially increase the diagnostic yield. Concerns over the safety of antiseizure medication reduction outside of a hospital environment have to date precluded increasing the yield of HVET studies through medication reduction but some have expressed the opinion that this may be excessively cautious.^24^ Where event frequency is very low, the practical upper limit of 6 days^25^ of remote recording without in‐person supervision for electrode‐related complications may ultimately mean that VT remains better undertaken in an inpatient environment. Conversely, those patients with high event frequency or concerns over hospital admission benefit from the flexibility offered by HVET and the possibility of event capture in an environment the patient may well prefer.

More generally, these observations regarding HVET fit in with wider findings regarding changes in clinical practice generally,^26^, ^27^ or epilepsy‐related work more specifically.^28^ Although we have focused here on HVET, we are equally interested in other aspects of remote diagnostic testing, and we suspect that some other modalities will also begin to thrive in the post‐pandemic world. Specifically, several technologies offer the prospect of longer‐term monitoring but also intrinsically share the characteristic of being intended for out‐of‐hospital use that made HVET robust to the disruptive qualities of the pandemic:

1. EEG monitoring technologies such as chronic subcutaneous or intracranial EEG^29^, ^30^ offer the opportunity to reliably detect seizure cycles and/or establish relative seizure burden.
2. Potentially more readily available in a wide variety of diagnostic and monitoring settings, remote video monitoring with human analysis (such as the vCreate platform adopted at Evelina Children's Hospital, Section 3.3.2) or partially automated video analysis with dedicated hardware (such as the system offered by NeuroEvent Labs^31^, ^32^).
3. Recording modalities that do not include EEG or video such as smartphone‐based reporting apps or wearable sensors^33^, ^34^ allow patients to report symptoms in near real time, or events to be semi‐automatically detected for establishing frequency and some risk‐related characteristics.

In summary, HVET continues to exhibit strengths that we and others have identified previously, specifically scalability and cost‐effectiveness. The COVID‐19 pandemic provided a momentous challenge to health care and elective diagnostic services such as VT, and the specific pressure on inpatient bed availability revealed that the “home” aspect of HVET provides additional resilience to this technique. The experience of our three centers has been that inpatient VT services failed in the face of pandemic‐induced pressures, whereas HVET proved sufficiently adaptable to not just survive the pandemic but to thrive and significantly expand in the unforeseen future.

## AUTHOR CONTRIBUTIONS

All authors contributed to the framework for the review and to writing of the manuscript. Collection and analysis of data from KCH was by DA, FB, and JSW. Collection and analysis of data from Evelina was by SG and JSW. Collection and analysis of data from Stratus was by JDS.

## CONFLICT OF INTEREST

JDS is employed as the Chief Medical Officer of Stratus. Stratus's primary service offering for patients is in‐home video‐EEG monitoring. No other authors report any conflicts of interest or disclosures relevant to this manuscript.
